# Daily-Life Walking Characteristics of Older Adults in Relation to Age, Sex, and Physical Function: the HUNT4 Trondheim 70+ Observational Study

**DOI:** 10.2196/75835

**Published:** 2025-12-04

**Authors:** Karoline Blix Grønvik, Anisoara Paraschiv-Ionescu, Gro Gujord Tangen, Øyvind Salvesen, Jorunn Lægdheim Helbostad, Nina Skjæret-Maroni, Beatrix Vereijken

**Affiliations:** 1 Department of Neuromedicine and Movement Science, Norwegian University of Science and Technology, Edvard Griegs Gate 8, Trondheim, 7030, Norway, 47 73592020; 2 Signal Processing Laboratory 5, Department of Electrical and Electronic Engineering, École Polytechnique Fédérale de Lausanne, Lausanne, Switzerland; 3 Department of Geriatric Medicine, Oslo University Hospital, Oslo, Norway; 4 Norwegian National Centre for Ageing and Health, Vestfold Hospital Trust, Tønsberg, Norway; 5 Department of Clinical and Molecular Medicine, Norwegian University of Science and Technology, Trondheim, Norway

**Keywords:** gait analysis, walking, daily life, real-world, older adults, Trøndelag Health Study, HUNT, Short Physical Performance Battery, SPPB, physical function

## Abstract

**Background:**

Knowledge about how older adults walk is crucial for the effective prevention and treatment of various mobility issues as well as treatment evaluation, but it is currently largely limited to laboratory-based measurements. Although laboratory-based data provide relevant information about what older adults can do under standardized conditions, they do not provide insight into how they actually walk in their daily life, a gap that needs to be addressed urgently.

**Objective:**

The objective of this study was to describe how older adults walk in daily life, in relation to age, sex, and level of physical function, using wearable sensor data from a large sample of older adults with a wide range of age and function from the HUNT4 Trondheim 70+ study.

**Methods:**

The current study is based on 1-week accelerometer data (Axivity AX3) from 1289 older adults (mean age 77.41, SD 6.06 years; age range 70‐105 years; n=705, 54.7% women). Physical function was assessed using the Short Physical Performance Battery (SPPB). To investigate the effect of age and SPPB score on gait metrics (daily number of steps, 95th percentile speed, mode speed, 95th percentile cadence, mode cadence, and maximum walking bout [WB] distance) for women and men, univariate gamma regression models with log link were used for each outcome measure, with age and SPPB score in separate models. Sex differences were investigated using Mann-Whitney *U* tests.

**Results:**

Older adults showed a large variation in how and how much they walked in daily life across age, sex, and physical function, particularly younger participants and those with better physical function. Most gait metrics decreased at an increasing rate with higher age, with men maintaining their levels up to higher ages than women. Poorer physical function led to an exponential or close-to-linear decrease in all gait metrics apart from habitual cadence, which remained stable up to a high age. Women had a lower daily number of steps, gait speed, and maximum distance but higher cadence than men (*P*<.001 for all). On average, 63% of all WBs lasted <10 seconds, corresponding to a median accumulated time of 99 (IQR 66‐128) minutes. For WBs lasting 10 to 30 seconds, 30 to 60 seconds, and >60 seconds, the median accumulated time was 105 (IQR 65‐154) minutes, 31 (IQR 18‐47) minutes, and 113 (IQR 37‐219) minutes, respectively.

**Conclusions:**

Daily-life walking performance was affected more by functional ability than by age itself, except for the highest ages, and differed significantly between sexes. Although most WBs were very short, the total accumulated walking time in WBs shorter than 30 seconds was longer than that in longer WBs. Future research can build upon our findings by considering both the impact of short WBs and relevant group and sex differences when implementing daily-life mobility assessment in both clinical studies and patient follow-up.

## Introduction

Mobility, the ability to move around physically, is one of the key features of independence for older adults, contributing to sustained participation and overall quality of life [1,2]. With increasing age, the prevalence of mobility problems increases, with almost a third of people over the age of 75 years having moderate to severe walking difficulties [3], leading to increased dependency on caregivers and assistive equipment [4]. Therefore, an in-depth understanding of how older adults walk can provide valuable insights into factors supporting mobility and independence in an aging population. A better understanding is pivotal for preventing reduced walking ability, thereby sustaining independence, lowering societal costs, and maximizing available resources [5], as well as maintaining their well-being [6].

Most of our current knowledge about how older adults walk is derived from laboratory-based gait assessments, which have provided us with detailed information about how older adults can perform under controlled, supervised conditions. As a result, gait speed has been identified as a predictor of survival [7] and disability [8] among older adults. Older adults who have a preferred gait speed below 1 m/s but are otherwise well functioning have a higher risk of adverse health-related outcomes than those with a higher habitual gait speed [9]. Furthermore, higher age, female sex, and lower physical function are associated with a lower gait speed [10,11]. Additionally, older adults who walk at a cadence of at least 100 steps per minute have a 21% lower risk of all-cause mortality than those with a lower step frequency [12].

However, laboratory-based gait assessments of what older adults can do under standardized conditions are not representative of what they actually do in daily life, and hence gait assessments in the laboratory and in daily life represent 2 different constructs of mobility [13-16]. Laboratory assessments may be biased by factors such as the “white coat effect” or the “Hawthorne effect,” which can lead to overperformance or underperformance or a general change in behavior due to the awareness of being observed [17]. In addition, laboratory assessments are limited to snapshots of a person’s gait, and gait speed measured in the laboratory differs significantly from gait speed in the real world [14,18]. Moreover, real-world cadence is significantly associated with number of falls per year, which has not been shown for cadence, gait speed, or distance measured in a laboratory setting [19]. Therefore, assessment outside the laboratory is needed to gain detailed knowledge of how older adults walk in a setting that reflects the complexity of their daily environments [1]. Such knowledge can provide essential complementary insights into critical factors to prevent functional decline and mobility reduction.

The last two decades have seen a rise and rapid improvement in wearable sensor technology that allows for continuous monitoring outside of a laboratory or clinical setting. These technological advances can provide researchers with fine-grained real-world data in a wide variety of mobility contexts. One of the first studies to use this technology was conducted by Orendurff and colleagues [20], who described daily-life walking patterns of healthy, employed adults. Since then, additional studies have investigated real-world walking using body-worn accelerometers [18,21-23]. Although these earlier studies provided the first insights into real-world gait, most of them had a small sample size [24], did not include the frailest and/or oldest adults [18,21,22], or used algorithms that were not validated specifically for an older population [22,23,25].

Recent developments in algorithms and analysis pipelines specifically developed for older adults and people experiencing mobility problems, along with improved sensor technology, make it possible to account for atypical walking patterns that can occur in these populations. These state-of-the-art algorithms have been shown to reliably estimate various digital mobility outcomes in a real-world environment, both for healthy older adults in general and for patients with mobility problems [26,27]. This paves the way for investigating how older adults walk in daily life in large study samples with large variations in age and level of function. Therefore, the objective of this study was to describe how older adults walk in daily life, in relation to age, sex, and level of physical function, using wearable sensor data from a large sample of older adults with a wide range of age and function from the HUNT4 Trondheim 70+ study.

## Methods

### Study Population

A total of 1745 adults aged 70 years or older participated in HUNT4 Trondheim 70+, a substudy in the fourth wave of the population-based Trøndelag Health Study (HUNT) in Norway [28]. Data collection took place from October 2018 to June 2019. To be able to recruit the oldest and frailest older adults as well, we offered a home visit to those who could not come to the field station. The majority of participants (1289/1745, 73.9%) were assessed at a field station, with the remaining participants assessed in their own homes (198/1745, 11.3%) or nursing homes (258/1745, 14.8%). Of the 1745 participants included in the Trondheim 70+ substudy, 1363 older adults (aged 70‐105 years) participated in activity monitoring.

### Ethical Considerations

This study was approved by the Regional Committee for Medical and Health Research Ethics (reference number 51105). The participants provided written informed consent. In cases where assessors or health care personnel in nursing homes considered a participant unable to consent, written informed consent was provided by the closest proxy. Participants could withdraw from the study at any moment without providing any reason. All collected data were deidentified to protect participant privacy and confidentiality. No compensation was provided apart from an offer of transportation to the field station if needed.

### Procedures

All participants in HUNT4 Trondheim 70+ underwent a standardized study protocol, including clinical tests, structured interviews, and self-reported questionnaires, which has been described in detail previously [29,30]. Height and body weight were measured as part of the clinical tests, and BMI (weight/height²) was calculated retrospectively. Cognitive function was assessed using the Montreal Cognitive Assessment (MoCA) [31], generating a score of 0 to 30, where higher scores indicated better cognitive performance. Physical function was assessed using the Short Physical Performance Battery (SPPB), consisting of 3 subtests targeting static balance (feet together and in semi-tandem and tandem stance), gait speed (4 m walk test), and leg strength (5 times sit-to-stand test). Each subtest was scored with a value between 0 and 4, adding up to a total score of 0 to 12 points, where higher scores indicated better performance [32].

After the clinical tests, participants were asked to wear 2 triaxial accelerometer devices (Axivity AX3), one on their thigh (centrally on the thigh, approximately 10 cm above the upper border of the patella) and one on the lower back (on L3, the third lumbar segment), for 7 days. Devices were configured using the OmGui software (version 1.0.0.43; Open Movement) and fixed on the skin using adhesive film (Opsite Flexifix; Smith & Nephew). Acceleration was measured at a frequency of 50 Hz and a range of ±8*g*. After the measurement period, devices were delivered back to the field station by the participant or a team member, where the data were downloaded using the OmGui software. Data from the lower back sensor were used in the analyses in this study.

### Data Processing

Of the 1363 participants who contributed activity monitoring data, 16 (1.2%) had a data collection registration error, 20 (1.5%) were missing data from the lower back sensor, and 31 (2.3%) had a data processing error. In addition, age and/or SPPB score were missing for 7 participants (0.5%). Data from the remaining 1289 participants (94.6%) were included in further analyses. First, the raw acceleration signal was resampled from 50 Hz to 100 Hz using interpolation [33], for compatibility with the subsequent algorithms. To distinguish between wearing and non-wearing of the sensor, we used an event-based algorithm that detects changes in the temperature signal from the Axivity AX3 device [34]. The number of days with accelerometry data ranged from 1 to 7. For 88.3% (1138/1289) of the participants, this value was 6 or 7 days; for 7.0% (90/1289), 4 or 5 days; and for less than 4.7% (61/1289), 3 days or less. Data processing was performed using MATLAB (version R2022a; MathWorks, Inc). The 456 participants excluded from the analyses (either because they never wore a body-fixed sensor or for the reasons mentioned above) were older (mean 82.2, SD 8.2 years versus mean 77.4, SD 6.1 years), had a lower MoCA score (mean 19.8, SD 6.9 versus 23.5, SD 4.2), and had a higher proportion of women (66.9% versus 54.7%) than those in the analyzed sample.

### Real-World Walking Analysis

The data processing pipeline included validated, top-ranked algorithms [26,27] associated with the following processing blocks: foot initial contact detection; walking bout (WB) detection; and estimation of cadence, step length, speed, and distance. The validity criteria were a 95% CI intraclass correlation coefficient threshold of ≥0.7 for performance metrics (eg, sensitivity, positive predictive value, and accuracy) and a relative error of <20%, as described by Micó-Amigo et al [27] and Kirk et al [26]. The *foot initial contact detection* algorithm used the preprocessed vertical acceleration signal. Preprocessing included detrending, low-pass filtering, numerical integration, and continuous wavelet transformation. The initial contact events were identified as the positive peaks between zero-crossings [27]. For *WB detection*, the norm of the triaxial acceleration signal was first detrended and low-pass filtered (finite impulse response, cutoff frequency=3.2 Hz); then, steps-related peaks in the acceleration signal were selected using adaptive thresholds for amplitude and peak-to-peak duration [35,36]. A WB was identified as a sequence of 2 or more consecutive steps (potentially including steps taken while turning), allowing 1.5 seconds plus the average step duration between consecutive steps [35], to ensure accurate estimation of gait metrics (eg, speed and cadence) and reduce bias. *WB duration* (s) was defined as the time interval from the first to the last foot initial contact of the WB. *Cadence* (steps/min) estimation was based on the foot initial contact detection algorithm and was calculated for each detected WB as the step frequency during the respective WB [27]. The *step length* (m) estimation algorithm was based on the inverted pendulum biomechanical model proposed by Zijlstra et al [27,37,38], using a correction factor in the model for impaired gait patterns. *Speed* (m/s) was estimated as the product of cadence and step length. For each WB, step length and speed were obtained as averaged values across all steps within the WB. Finally, *WB distance* (m) was estimated as the product of WB duration and WB speed.

### Data Aggregation and Gait Outcome Metrics

As the parameters characterizing real-world walking were estimated for each detected WB throughout the monitoring period, a data aggregation procedure was required to summarize these WB parameters as gait outcome variables for each participant before subsequent statistical analysis. To this end, for each participant, the WBs and the respective parameters were aggregated from (1) all WBs across valid wear days (daily aggregation) or (2) all WBs across the entire measurement period (weekly aggregation). *Daily number of steps* was obtained by summing the steps within all detected WBs across all valid days and then averaging across all valid days. Valid days were defined as days having at least 18 hours of detected wear time. The weekly aggregation procedure was based on various statistical metrics that describe the distribution of walking parameters across pooled WBs. The *within-subject mode* values of cadence and speed were used to represent habitual walking. The *within-subject 95th percentile* values of cadence and speed, referred to as fast cadence and fast walking speed, respectively, were used to represent walking capacity. *Maximum WB distance* was defined as the longest distance covered in a single WB across all measurement days per participant. Additional gait outcome variables derived for weekly aggregated WBs were as follows: (1) *WB duration distribution,* defined as the proportion of WBs in each of the 4 duration categories (≤10 s, >10‐30 s, >30‐60 s, >60 s), and (2) *time in WB duration categories,* defined as the accumulated time in each of the 4 WB duration categories.

### Cohort Stratification Criteria

Participants were categorized on the basis of age and SPPB score to facilitate the graphical representation of group variance. They were divided into 6 groups according to age (70.0‐74.9, 75.0‐79.9, 80.0‐84.9, 85.0‐89.9, 90.0‐94.9, and ≥95.0 years) and 3 groups according to SPPB score (range=0‐12 points; high=12‐10 points, moderate=9‐7 points, and low=6‐0 points) [39].

### Statistical Analysis

Statistical analyses were performed using R software (version 4.4.3; R Foundation for Statistical Computing) and RStudio (version 2024.04.1+748 Chocolate Cosmos; Posit Software, PBC). Participant characteristics were summarized using mean and SD for continuous variables and frequencies and percentages for categorical variables. The normality distribution of dependent variables was assessed visually using histograms and QQ plots and statistically using the Shapiro-Wilk test and skewness and kurtosis tests. To assess whether participant characteristics and gait metrics differed significantly by sex, we applied the nonparametric Mann-Whitney *U* test. A *χ*^2^ test of independence was used to test whether the distribution of SPPB score differed between sexes.

To investigate the effect of age and SPPB score on gait metrics (daily number of steps, 95th percentile speed, mode speed, 95th percentile cadence, mode cadence, and maximum WB distance), univariate gamma regression models with log link were used for each outcome measure (one aggregated value per participant), with age and SPPB score in separate models. Models using gamma family and log link were chosen after inspecting residual distributions and model fit to account for the data distribution, and age and SPPB score were treated as continuous variables. The SPPB score was reversed to use the maximum score of 12 points as the reference. To prevent disproportionate influence on the results, single representatives of a specific age (2 women aged 97 and 99 years, respectively, and 2 men aged 93 and 100 years, respectively) were excluded from the gamma regression models. A power law relationship was observed between several log gait metrics and age or SPPB score. Therefore, the exponent providing the best fit was determined using maximum likelihood estimation. When the Akaike information criterion did not improve, the exponent was fixed at 1. Regression analyses were performed separately for women and men.

The relationship between each gait metric and age was assessed using gamma regression models assuming


$$gait\;metric=exp\left(\beta_{0}+\;\beta_{1}{\left(age-70\right)}^{\beta_{p}}\right)\epsilon$$

with a gamma distribution with mean 1 and variance ϕ for the error term ϵ.

The relationship between each gait metric and the SPPB score was assessed using gamma regression models assuming


$$gait\;metric=exp\left(\beta_{0}+\;\beta_{1}{\left(12-score\right)}^{\beta_{p}}\right)\epsilon$$

with a gamma distribution with mean 1 and variance ϕ for the error term ϵ.

## Results

A total of 1289 participants (n=705, 54.7% women) were included in the analyses. The mean age of the participants was 77.41 (SD 6.06) years, ranging from 70 to 105 years. Significant differences were found between sexes in age, height, weight, BMI, and overall SPPB score (see Table 1 for the respective *P* values). Furthermore, the distribution of SPPB categories differed significantly between women and men (*χ*²_2_=8.34, *P*=.02), with a higher proportion of men in the high SPPB score group. Additional participant characteristics and group frequencies are presented in Table 1.

**Table 1.** Participant characteristics and group frequencies.

	Total	Women	Men	*P* value^a^
Participants, n (%)	1289 (100)	705 (54.7)	584 (45.3)	—^b^
Age (years), mean (SD) [range]	77.41 (6.06) [70.1‐105.4]	77.96 (6.45)	76.74 (5.49)	.005
Height (cm), mean (SD) [range]	168.80 (9.26) [141-199]	162.70 (6.36)	176.10 (6.49)	<.001
Weight (kg), mean (SD) [range]	76.20 (14.81) [36.4‐195.7]	69.90 (12.72)	83.74 (13.56)	<.001
BMI (kg/m²), mean (SD) [range]	26.71 (4.43) [13.4‐65.4]	26.45 (4.68)	27.02 (4.09)	.002
MoCA^c^ score, mean (SD) [range]	23.50 (4.19) [3-30]	23.56 (4.45)	23.43 (3.87)	.10
SPPB^d^ score, mean (SD) [range]	9.96 (2.94) [0‐12]	9.71 (3.09)	10.27 (2.71)	<.001
Group frequency, n (%)				
SPPB^d^ category				.02
High	923 (71.6)	482 (68.4)	441 (75.5)	
Moderate	203 (15.8)	121 (17.2)	82 (14)	
Low	163 (12.7)	102 (14.5)	61 (10.6)	
Age category (years)				—
70.0‐74.9	588 (45.6)	310 (44)	278 (47.6)	
75.0‐79.9	341 (26.4)	174 (24.7)	167 (28.6)	
80.0‐84.9	188 (14.6)	110 (15.6)	78 (13.4)	
85.0‐89.9	104 (8.1)	65 (9.2)	39 (6.7)	
90.0‐94.9	54 (4.2)	35 (5)	19 (3.2)	
≥95.0	14 (1.1)	11 (1.6)	3 (0.5)	

a Derived from Mann-Whitney *U* tests and *χ*2 test of independence.

b Not applicable.

c MoCA: the Montreal Cognitive Assessment. Generates a score of 0-30, where higher scores indicate better cognitive performance.

d SPPB: Short Physical Performance Battery. Generates a score of 0-12, where higher scores indicate better physical performance. high=12-10 points, moderate=9-7 points, low=6-0 points.

### Number and Characteristics of WBs

The mean number of daily WBs was 253.46 (SD 123.63), ranging from 2.25 to 865.5 WBs. There was no significant difference between women and men in number of WBs (*P*=.47). Across participants, on average, 63% of all WBs had a duration of ≤10 seconds, while only 6% of all WBs lasted >30 seconds, independent of age, sex, and physical function. The median total accumulated time spent walking in WBs of <10 seconds was 99 (IQR 66‐128) minutes, and it was 105 (IQR 65‐154) minutes for WBs lasting between 10 and 30 seconds. Furthermore, WBs lasting between 30 and 60 seconds and >60 seconds had a median accumulated time of 31 (IQR 18‐47) minutes and 113 (IQR 37‐219) minutes, respectively. Of the 1289 participants, only 1 participant (0.1%) did not have WBs of >10 seconds, 35 participants (2.7%) did not have WBs of >30 seconds, and 83 participants (6.4%) did not have WBs of >60 seconds.

For women, WBs lasting <10 seconds accounted for 66.3% of all WBs, adding up to a median total accumulated time of 101 (IQR 65‐133) minutes. In contrast, men had 59.2% WBs of <10 seconds, with a median total accumulated time of 96 (IQR 66‐125) minutes. Bouts lasting between 10 and 30 seconds accounted for 28% of WBs for women and 33.6% for men, with a median total accumulated time of 92 (IQR 58‐137) minutes and 120 (IQR 78‐178) minutes, respectively. Furthermore, 2.7% of women’s and 4.0% of men’s WBs lasted between 30 and 60 seconds, with a median total accumulated time of 27 (IQR 14‐39) minutes for women and 37 (IQR 24‐55) minutes for men. Only 2.3% of women’s WBs and 2.8% of men’s WBs lasted longer than 60 seconds, with a median total accumulated time of 96 (IQR 29‐210) minutes and 134 (IQR 54‐227) minutes, respectively.

### Daily Number of Steps

**Figure 1.**
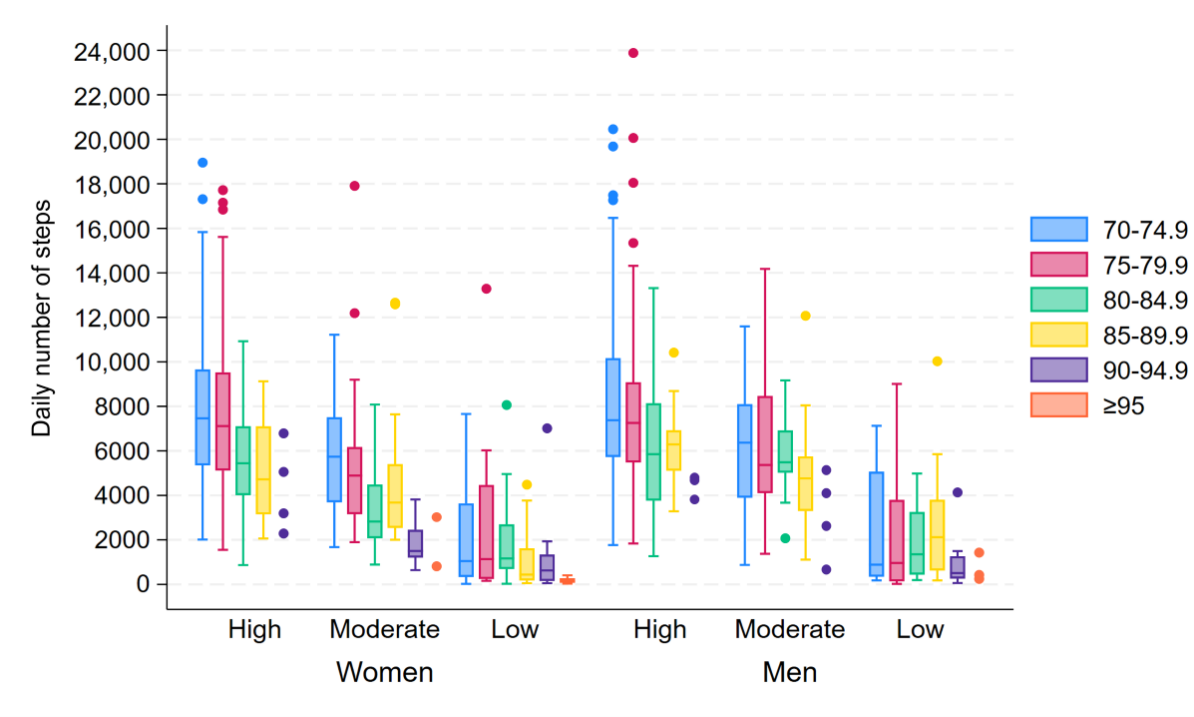
Box plots of daily number of steps, showing the median and IQR (whiskers) of daily steps across all valid days, stratified by age group (years), sex, and SPPB score group (high=12‐10 points, moderate=9‐7 points, low=6‐0 points). For groups with fewer than 5 participants, individual data points are displayed. SPPB: Short Physical Performance Battery.

The average number of steps per day varied widely, particularly in younger age groups and higher SPPB score groups (Figure 1). Overall, women took significantly fewer daily steps than men, with the medians being 5704.5 (IQR 3441.6‐8264.8) and 6504.5 (IQR 4442.3‐8837.0), respectively (*P*<.001). Furthermore, participants with higher age and lower SPPB scores had fewer daily steps.

The daily number of steps decreased with higher age and lower SPPB scores for both women and men. For women, results from the gamma regression analyses examining the effect of age on the daily number of steps predicted a gradual decrease until approximately 77 years of age and then a decrease at an accelerating rate with advancing age (detailed results from all gamma regression models are presented in Tables S1-S4 in Multimedia Appendix 1). In comparison, men were predicted to show a slower decrease in the daily number of steps up to approximately 80 years of age, after which the rate of decrease became more prominent with increasing age, eventually exceeding the rate of decrease for women. The predicted decrease in the daily number of steps as age increased from 70 to 71 years was 0.06% for women and 0.006% for men, with the rate of decrease accelerating to 5.6% and 3.6% with an increase in age from 80 to 81 years and 14.8% and 15.2% with an increase in age from 90 to 91 years for women and men, respectively.

Likewise, the gamma regression analyses predicted an accelerating rate of decrease in the daily number of steps with poorer SPPB scores, although the acceleration was less prominent than that observed for age. Women showed a larger decrease with decreasing SPPB scores already in the highest score range, while men showed a slower decrease in the daily number of steps in the higher SPPB score range before the rate of decrease exceeded women’s rate of decrease in the lowest SPPB score range. The predicted decrease in the daily number of steps with a reduction in SPPB score from 12 to 11 points was 5.45% for women and 1.19% for men, with the decrease escalating to 23.4% and 20.9% with a score reduction from 6 to 5 points and 34.6% and 44.9% with a score reduction from 1 to 0 points, respectively.

### Walking Speed

**Figure 2.**
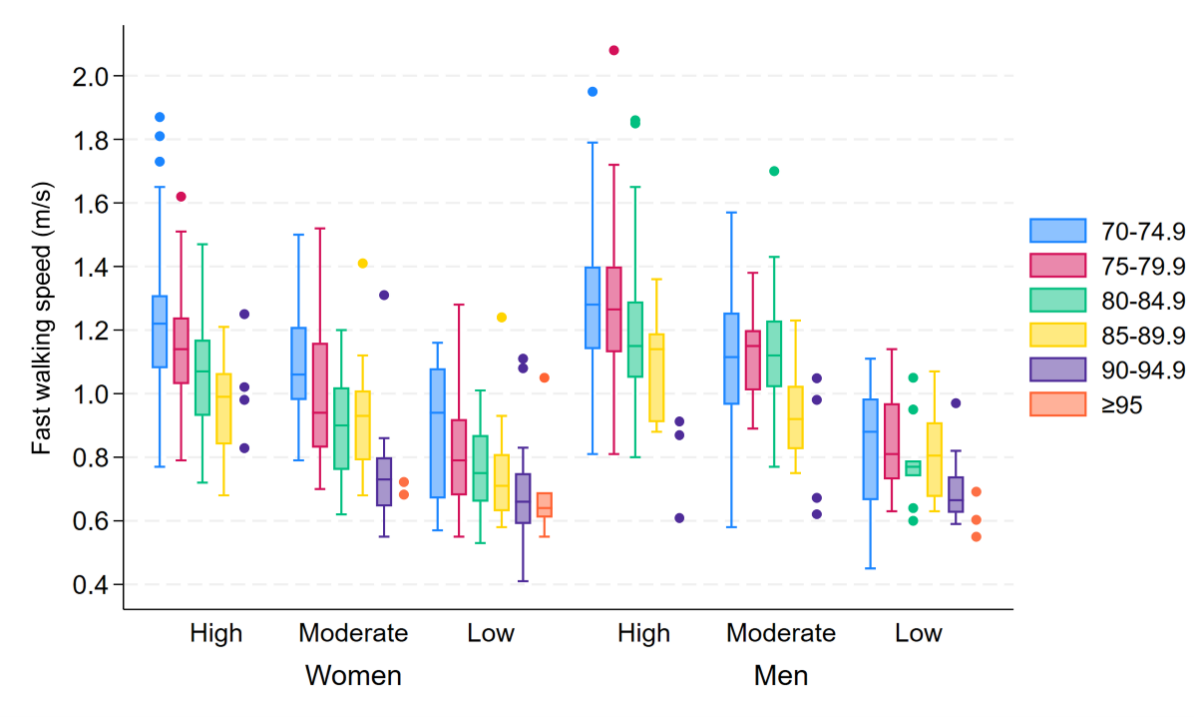
Box plots of fast walking speed, showing the between-subject median and IQR of the within-subject 95th percentiles of gait speed (m/s), stratified by age group (years), sex, and SPPB score group (high=12‐10 points, moderate=9‐7 points, low=6‐0 points). For groups with fewer than 5 participants, individual data points are displayed. SPPB: Short Physical Performance Battery.

**Figure 3.**
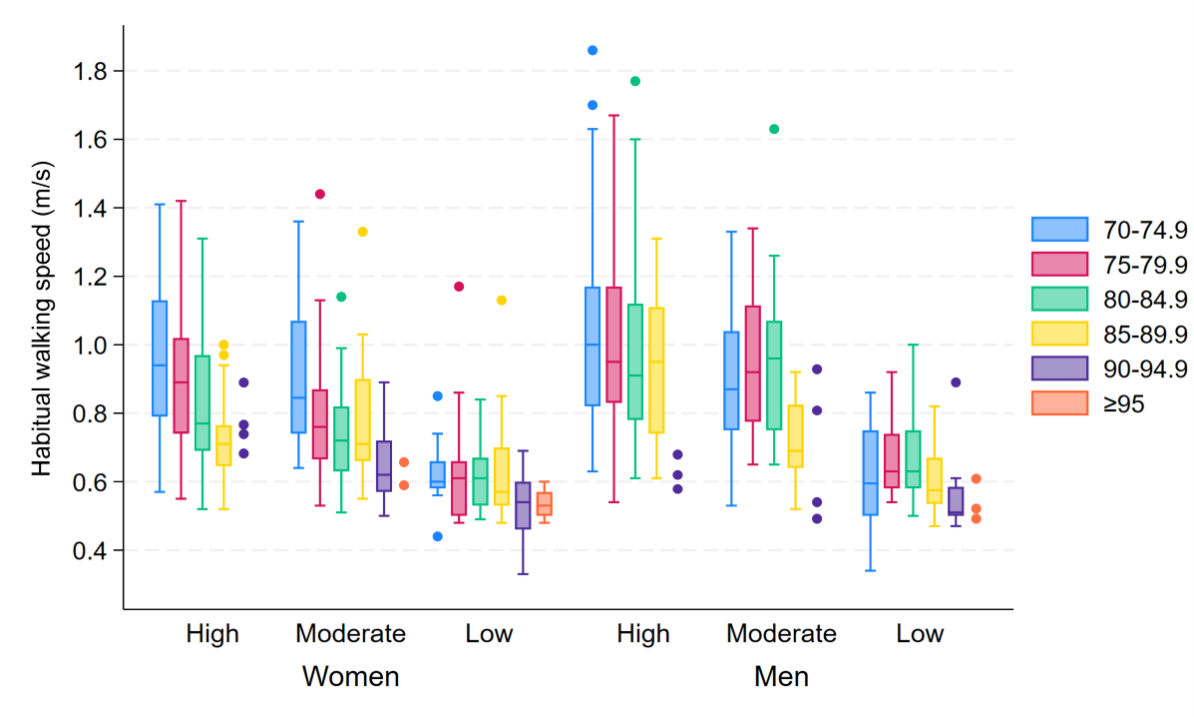
Box plots of habitual walking speed, showing the between-subject median and IQR of the within-subject modes of gait speed (m/s), stratified by age group (years), sex, and SPPB score group (high=12‐10 points, moderate=9‐7 points, low=6‐0 points). For groups with fewer than 5 participants, individual data points are displayed. SPPB: Short Physical Performance Battery.

Similar to the findings regarding the daily step count, there were large variations in both fast and habitual walking speeds, particularly in the younger age groups and higher SPPB score groups (see Figure 2 for fast speed and Figure 3 for habitual speed). Furthermore, both fast and habitual speeds decreased with increasing age and poorer SPPB scores, and women generally walked slower than men. For women and men, the median fast walking speed was 1.08 (IQR 0.91‐1.24) m/s and 1.19 (IQR 1.03‐1.35) m/s, respectively (*P*<.001), and the median habitual walking speed was 0.80 (IQR 0.68‐1.01) m/s and 0.91 (IQR 0.76‐1.12) m/s, respectively (*P*<.001).

Overall, both fast and habitual walking speeds were predicted to decrease at an accelerating rate with age. However, both fast and habitual speeds were predicted to decrease evenly with age for women but to have a slow initial decline and steeper decline with higher age for men, similar to the predicted decline in daily number of steps with age. In other words, women experienced a more prominent decline at an earlier age than men, the latter showing a slower rate of decline until approximately 80 years of age. With an increase in age from 70 to 71 years, women’s fast and habitual speeds were predicted to decrease by 0.97% and 1.13%, respectively, with the decrease in fast and habitual speeds accelerating to 2.16% and 2.20% with an increase from 80 to 81 years and 2.55% and 2.51% with an increase from 90 to 91 years, respectively. In contrast, with an increase in age from 70 to 71 years, men were predicted to have an initial decrease of only 0.02% for fast speed and 0.01% for habitual speeds, with the decline accelerating to 1.88% and 1.69% with an increase from 80 to 81 years and 5.12% and 5.42% with an increase from 90 to 91 years, respectively.

With a decrease in SPPB score, an exponential decrease was predicted in both fast and habitual speeds for both sexes. In other words, results indicated a rapid initial decrease in gait speed as the score decreased from 12 points, with a diminishing rate of decrease in speed further down the score scale. Per 1-point decrement in SPPB score, women’s fast speed and habitual speed were predicted to decrease by 5.06% and 5.15%, respectively. For men, the predicted decrease per 1-point decrement in SPPB score was 5.44% for fast speed and 5.29% for habitual speed.

### Cadence

**Figure 4.**
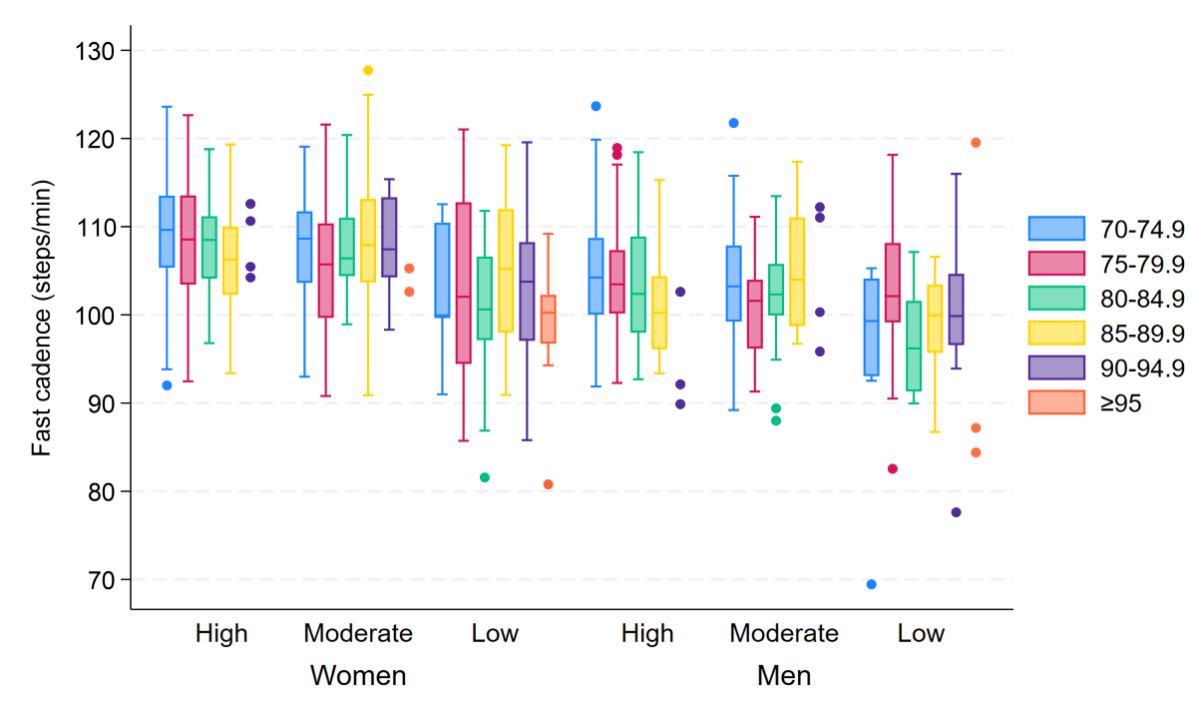
Box plots of fast cadence, showing the between-subject median and IQR of the within-subject 95th percentile of cadence (steps/min), stratified by age group (years), sex, and SPPB score group (high=12‐10 points, moderate=9‐7 points, low=6‐0 points). For groups with fewer than 5 participants, individual data points are displayed. SPPB: Short Physical Performance Battery.

**Figure 5.**
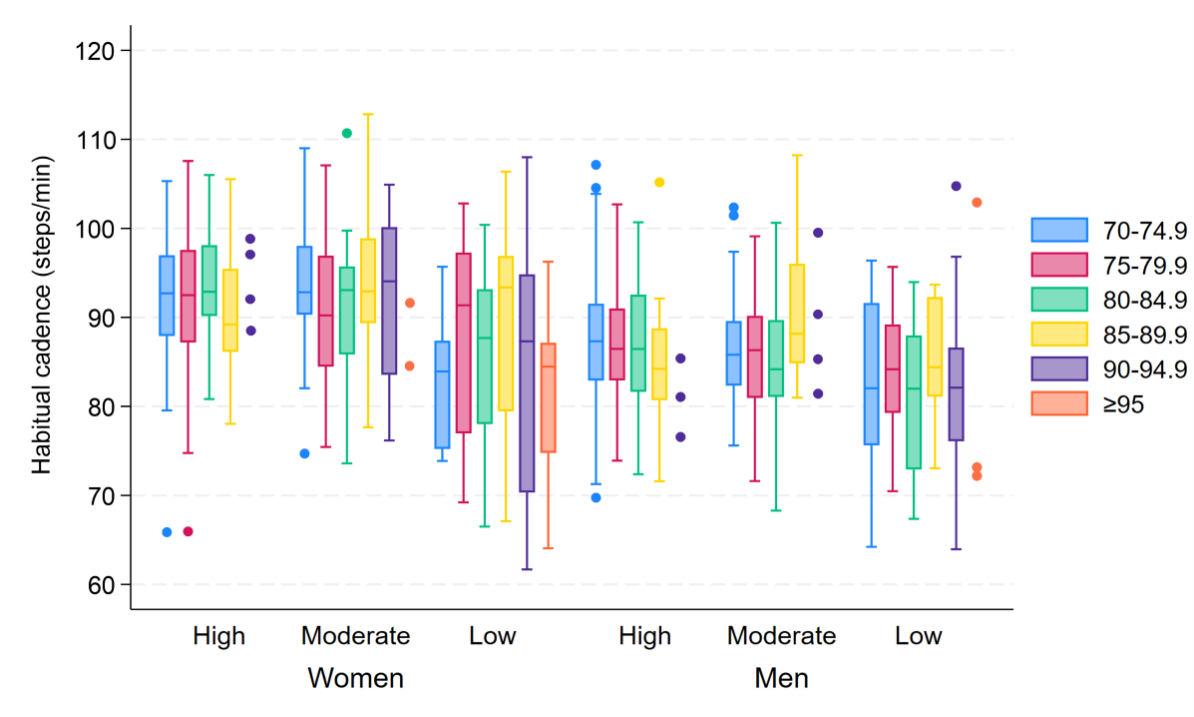
Box plots of habitual cadence, showing the between-subject median and IQR of the within-subject mode of cadence (steps/min), stratified by age group (years), sex, and SPPB score group (high=12‐10 points, moderate=9‐7 points, low=6‐0 points). For groups with fewer than 5 participants, individual data points are displayed. SPPB: Short Physical Performance Battery.

Although there was an overall large variation in cadence within all age and SPPB groups, both fast and habitual cadence remained stable up to higher ages than daily number of steps and speed. Furthermore, women had an overall higher cadence compared to men: median fast cadence 107.9 (IQR 103.3‐112.2) steps/min and 103.3 (IQR 99.1‐107.6) steps/min, respectively (*P*<.001; Figure 4), and median habitual cadence 92.1 (IQR 86.8‐97) steps/min and 86.4 (IQR 82.4‐91.1) steps/min, respectively (*P*<.001; Figure 5).

With increasing age, fast cadence was predicted to decrease slowly for both women and men, with a decrease of 0.20% and 0.25% per 1-year increase in age, respectively. Results indicated virtually no decrease in habitual cadence until the age of approximately 85 to 90 years for both women and men, followed by a steep decline after the age of 90 years. With an increase in age from 70 to 71 years, habitual cadence decreased by <0.001% for both women and men, with the rate of decrease accelerating to 0.04% and 0.003% with an increase in age from 80 to 81 years and 0.88% and 1.18% with an increase in age from 90 to 91 years, respectively.

With a decrease in SPPB score, fast cadence was predicted to decrease exponentially for both women and men, with an overall larger effect in women than in men. For every 1-point decrement in the SPPB score, fast cadence was predicted to decrease by 0.69% for women and 0.50% for men. However, habitual cadence was predicted to decrease at an accelerating rate for every 1-point decrement in SPPB score for both sexes. In other words, the habitual cadence remained more stable at higher SPPB scores before decreasing at an accelerating rate toward the lowest SPPB scores, particularly for men. The rate of decrease in habitual cadence with a reduction in SPPB score from 12 to 11 points was 0.18% for women and 0.02% for men, with the rate of decrease accelerating to 0.98% and 0.62% for a score reduction from 6 to 5 points and 1.64% and 1.77% for a reduction from 1 to 0 points, respectively.

### Maximum WB Distance

**Figure 6.**
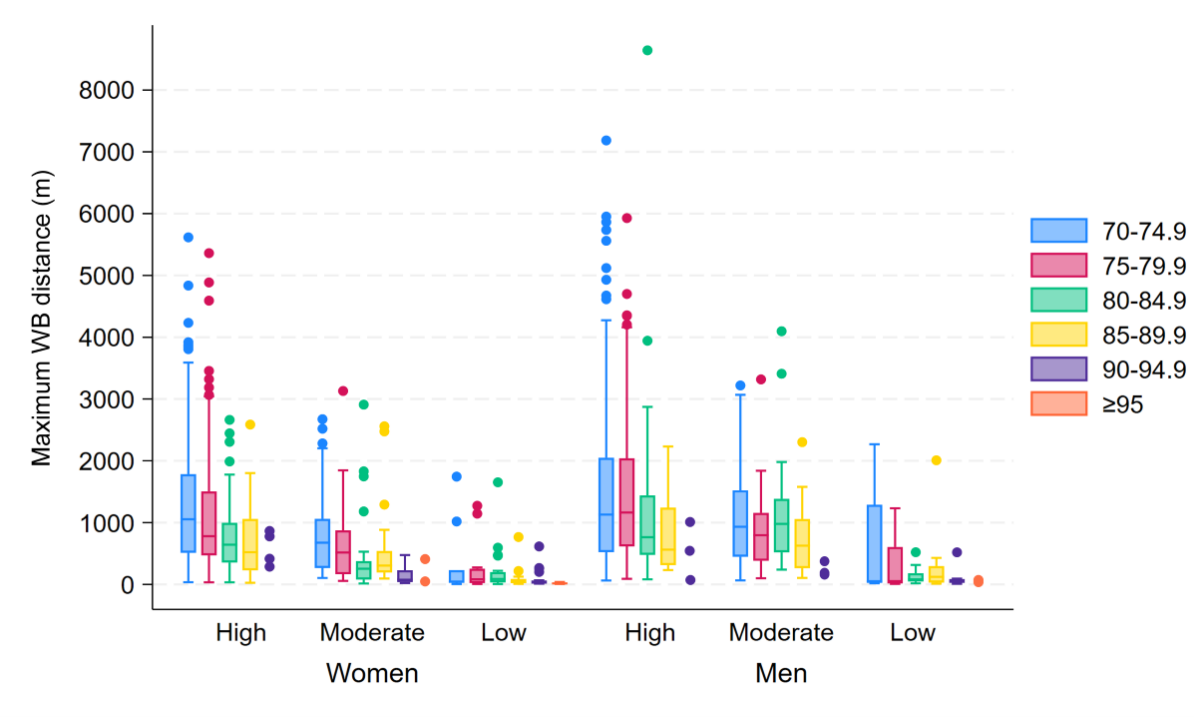
Box plots of maximum walking bout (WB) distance, showing the between-subject median and IQR of the within-subject maximum WB distance (m), stratified by age group (years), sex, and SPPB score group (high=12‐10 points, moderate=9‐7 points, low=6‐0 points). For groups with fewer than 5 participants, individual data points are displayed. SPPB: Short Physical Performance Battery.

The longest distance covered in a single WB showed a pattern similar to that observed for daily number of steps and speed, with large variations, particularly in the younger age groups and in the higher SPPB score groups (Figure 6). Additionally, individuals with higher SPPB scores and younger age showed an overall longer maximum WB distance, and women generally covered a shorter maximum WB distance than men, with the median (IQR) values being 615.4 (229.6‐1332.6) m and 869.8 (365.6‐1724.1) m, respectively (*P*<.001).

In women, the maximum WB distance was predicted to initially decrease evenly, before stabilizing at a median between 10 and 40 m after the age of 90 years. Interestingly, although men’s maximum WB distance initially decreased slowly, the rate of change subsequently accelerated from the age of around 80 years, ultimately leading to men having a steeper decrease at older ages than women. However, men’s maximum WB distance did not fall below a median of 40 m until the age of 93 years. The predicted decrease in maximum WB distance with an increase in age from 70 to 71 years was 0.28% for women and 0.01% for men, with the decline accelerating to 8.14% and 5.13% with an increase in age from 80 to 81 years and 16.47% and 20.29% with an increase in age from 90 to 91 years, respectively.

As Figure 6 shows, both women and men had lower maximum WB distances with lower SPPB scores. For women, a relatively even decrease was predicted with lower SPPB scores, while men were predicted to maintain a higher maximum WB distance to a larger degree despite lower SPPB scores, dropping to low WB distances only in the highest age groups. The gamma regression analysis predicted a decrease in maximum WB distance of 11.74% for women and 2.97% for men when the SPPB score decreased from 12 to 11 points, with the rate of decrease accelerating to 28.56% and 26.26% with a decrease in score from 6 to 5 points and 36.11% and 46.16% with a decrease in score from 1 to 0 points, respectively.

## Discussion

### Principal Findings

This study investigated the daily-life walking performance of older adults, focusing on age, sex, and level of physical function. With comprehensive data from a large sample covering a wide range of age and function, our results bring valuable new insights into how older adults walk outside a laboratory or clinical setting. Overall, there was a large variation between older adults in how and how much they walked in daily life, particularly among younger participants and those with better physical function. Despite this variability, the results revealed clear patterns in daily-life walking performance and mobility among older adults, with notable differences between men and women. While women generally had a higher cadence and shorter WBs, men had a longer maximum WB distance. Across both sexes, higher age and lower physical function were consistently associated with reductions in the number of daily steps, walking speed, and maximum WB distance. These declines intensified at an advanced age, highlighting the nonlinear decline in mobility in later life. Surprisingly, older adults maintained their habitual cadence up to high ages. Finally, the total walking time in WBs shorter than 30 seconds exceeded that in bouts longer than 30 seconds, indicating that, on average, older adults accumulate the majority of their walking time in short WBs.

### Daily-Life Walking in Relation to Age, Sex, and Physical Function

Consistent with earlier research, daily-life walking predominantly consisted of very short WBs [20,40]. Our results built further on this finding by highlighting interesting sex differences in walking behavior. Women tended to engage in shorter WBs and accumulated more time in these brief episodes, while men generally walked more and accumulated more time in longer bouts. Although women generally had fewer daily steps than men, this was not the case in the youngest age group, which aligns with previous findings in the age groups 45 to 79 years [21] and 60 to 79 years [22]. Across all ages, women walked at slower speeds, consistent with previous findings from both in-laboratory [11,41,42] and daily life measurements [21,22,43]. Furthermore, the maximum WB distance was shorter for women, which stands in contrast to the findings of Kiselev et al [22], who reported longer maximum WB distances for women. This discrepancy may be partly due to the younger cohort (60‐79 years) in Kiselev et al [22] than in this study (70-105 years). Despite walking slower, women maintained a higher cadence than men, a trend also observed in earlier research [42,43].

Interestingly, age-related changes in daily-life walking behavior were not linear. Daily steps, walking speed, and maximum WB distance declined gradually at first, followed by a more pronounced reduction with advancing age. Although women showed signs of decline earlier, men experienced a steeper drop in later years, eventually resulting in a higher rate of change at the highest ages. These findings go beyond patterns found in earlier studies that used linear models, such as Kiselev et al [22]. Our findings align with those of Ko et al [44], who observed increasingly pronounced declines in gait speed with age in laboratory settings, and of Obuchi et al [43], who reported similar sex-specific trends in daily-life walking in Japanese adults. Notably, habitual cadence remained stable up to a high age, with both sexes largely maintaining their habitual cadence until 85 to 90 years of age, after which the decrease accelerated drastically. Women simultaneously showed a decline in walking speed, suggesting a reduction in their stride length.

Physical function, assessed using the SPPB, emerged as a stronger influence than age itself on daily-life walking behavior. Except for habitual cadence, which remained relatively stable across most levels of physical function, declines in the remaining gait metrics were more pronounced with lower SPPB scores, indicating that functional ability plays a central role in maintaining mobility. This supports previous claims that gait changes cannot be fully explained by age, sex, height, or BMI alone [22] and highlights the importance of including an assessment of physical function. In contrast to our findings, van Gameren et al [13] found weaker associations between SPPB and walking metrics, potentially due to their exclusion of short WBs, which comprised over 60% of our dataset.

Although the SPPB is frequently used as a measure of physical function in older adults [10,16,30], it has known ceiling effects among those with high-level functioning [45,46], which may conceal early, more subtle declines in physical function. Hence, future research should strive to incorporate more sensitive assessment tools capable of distinguishing performance within this well-functioning subgroup.

### Methodological Considerations

This study has 2 key methodological strengths: a robust sample and validated algorithms to estimate daily-life gait metrics. The HUNT4 Trondheim 70+ cohort comprises a large and diverse group of older adults, spanning a broad range in age and function, with near-equal sex distribution. Furthermore, the vast majority of participants contributed 6 or 7 days of accelerometry data, likely due to the use of body-fixed sensors secured with adhesive tape, minimizing participant interference and ensuring consistent data collection. The digital mobility outcomes in our study were derived using algorithms previously validated in older adults and various clinical populations [26,27], including frail individuals recovering from hip fractures [47]. Although these algorithms were developed to be device agnostic, we used a different yet similar device (the AX3 instead of the AX6) as well as a different sampling frequency, requiring resampling of our data. In the original validation work, algorithm performance improved further with the exclusion of WBs shorter than 10 seconds and among participants with better physical function [27,47]. However, as these short bouts represented the majority of walking behavior in our cohort, they were retained in the current analyses. The exclusion of breaks within WBs strengthened the estimation of speed and cadence but may have led to an underestimation of walking duration and distance when short breaks segmented a walking period into multiple bouts. However, the inclusion of short WBs and exclusion of breaks offers a close description of walking behavior in older adults as it occurs in their daily lives, which is frequently interrupted by environmental factors and activity transitions. Future research should explore whether and how the frequency and duration of breaks influence the estimations of walking duration and distance in naturalistic settings. Furthermore, future research should investigate how older adults walk in relation to other potentially influencing factors such as cognitive function, comorbidities, environmental conditions, and psychosocial factors.

### Conclusion

Based on a large study sample with a wide range of age and function, this study provides new insights into how older adults walk in daily life. First of all, there was a persistent large variation in how older adults walk both across and within age groups up to the highest ages. Furthermore, although gait metrics generally decreased with increased age and decreased physical function, changes were largely nonlinear and showed relevant differences between men and women, with men maintaining how they walked in daily life up to a higher age than women but with accelerated change at the highest ages. Surprisingly, habitual cadence remained remarkably stable compared to other gait characteristics in both men and women throughout most ages and levels of physical function. Moreover, although most WBs were very short, the total accumulated walking time in bouts shorter than 30 seconds exceeded that in longer WBs. Finally, daily-life walking performance was affected more by changes in functional ability than by increased age per se. Future research can build upon these findings by implementing daily-life mobility assessment in both clinical studies and patient follow-up, to monitor changes in gait that may point to underlying changes in health condition and function, while considering the impact of short WBs and relevant group and sex differences in how older adults walk in daily life.

## Supplementary material

10.2196/75835 Multimedia Appendix 1 Results of gamma regression models with log link for each gait metric with age or Short Physical Performance Battery score

## Abbreviations

HUNT: Trøndelag Health Study
MoCA: Montreal Cognitive Assessment
SPPB: Short Physical Performance Battery
WB: walking bout
