# Supplementary material for: Daily-Life Walking Characteristics of Older Adults in Relation to Age, Sex, and Physical Function: the HUNT4 Trondheim 70+ Observational Study
Source: JMIR Aging. 2025 Dec 4;8:e75835. doi: 10.2196/75835 (PMC12677731; doi:10.2196/75835)
Supplement: Multimedia Appendix 1 [file aging-v8-e75835-s001.docx]

Table S1. Results from gamma regression with log link for each gait metric with age for women.

| **Dependent variable** | **β_0_ (Intercept)** | **β_1_ (Coefficient)** | **β_p_^a^** | ***P*-value** | **Disp. Param.^b^** |
| --- | --- | --- | --- | --- | --- |
| Daily number of steps | 8.9015 | -.0006 | 2.54 | <.001 | 0.3597 |
| Fast speed | 0.1959 | -.0098 | 1.25 | <.001 | 0.0346 |
| Habitual speed | 0.0035 | -.0219 | 1 | <.001 | 0.0526 |
| Fast cadence | 4.6952 | -.0020 | 1 | <.001 | 0.0038 |
| Habitual cadence | 4.5222 | -1.0974×10^−9^ | 5.69 | <.001 | 0.0069 |
| Maximum WB distance | 7.1058 | -.0028 | 2.12 | <.001 | 1.0237 |

^a^β_p_: The exponent providing the best fit. When the AIC did not improve, the exponent was fixed at one.

^b^Disp. Param.: Dispersion Parameter.

Table S2. Results from gamma regression with log link for each gait metric with age for men.

| **Dependent variable** | **β_0_ (Intercept)** | **β_1_ (Coefficient)** | **β_p_^a^** | ***P*-value** | **Disp. Param.^b^** |
| --- | --- | --- | --- | --- | --- |
| Daily number of steps | 8.9186 | -.0001 | 3.23 | <.001 | 0.2727 |
| Fast speed | 0.2221 | -.0002 | 2.52 | <.001 | 0.0354 |
| Habitual speed | -0.0086 | -.0001 | 2.78 | <.001 | 0.0624 |
| Fast cadence | 4.6540 | -.0025 | 1 | <.001 | 0.0039 |
| Habitual cadence | 4.4630 | -1.31×10^−15^ | 10.11 | <.001 | 0.0062 |
| Maximum WB distance | 7.2324 | -.0001 | 3.18 | <.001 | 0.9015 |

^a^β_p_: The exponent providing the best fit. When the AIC did not improve, the exponent was fixed at one.

^b^Disp. Param.: Dispersion Parameter.

Table S3. Results from gamma regression with log link for each gait metric with SPPB score for women.

| **Dependent variable** | **β_0_ (Intercept)** | **β_1_ (Coefficient)** | **β_p_^a^** | ***P*-value** | **Disp. Param.^b^** |
| --- | --- | --- | --- | --- | --- |
| Daily number of steps | 8.9355 | -.0561 | 1.63 | <.001 | 0.3789 |
| Fast speed | 0.1694 | -.0520 | 1 | <.001 | 0.0308 |
| Habitual speed | -0.0526 | -.0528 | 1 | <.001 | 0.0466 |
| Fast cadence | 4.6949 | -.0070 | 1 | <.001 | 0.0037 |
| Habitual cadence | 4.5298 | -.0018 | 1.70 | <.001 | 0.0069 |
| Maximum WB distance | 7.1360 | -.1249 | 1.39 | <.001 | 1.2428 |

^a^β_p_: The exponent providing the best fit. When the AIC did not improve, the exponent was fixed at one.

^b^Disp. Param.: Dispersion Parameter.

Table S4. Results from gamma regression with log link for each gait metric with SPPB score for men.

| **Dependent variable** | **β_0_ (Intercept)** | **β_1_ (Coefficient)** | **β_p_^a^** | ***P*-value** | **Disp. Param.^b^** |
| --- | --- | --- | --- | --- | --- |
| Daily number of steps | 8.9379 | -.0120 | 2.26 | <.001 | 0.2904 |
| Fast speed | 0.2518 | -.0560 | 1 | <.001 | 0.0285 |
| Habitual speed | 0.0253 | -.0543 | 1 | <.001 | 0.0545 |
| Fast cadence | 4.6463 | -.0050 | 1 | <.001 | 0.0040 |
| Habitual cadence | 4.4670 | -.0002 | 2.42 | <.001 | 0.0063 |
| Maximum WB distance | 7.2633 | -.0302 | 1.96 | <.001 | 0.9932 |

^a^β_p_: The exponent providing the best fit. When the AIC did not improve, the exponent was fixed at one.

^b^Disp. Param.: Dispersion Parameter.
